# Effect of PDLA and Amide Compounds as Mixed Nucleating Agents on Crystallization Behaviors of Poly (l-lactic Acid)

**DOI:** 10.3390/ma11071139

**Published:** 2018-07-05

**Authors:** Thanawat Khwanpipat, Manus Seadan, Supakij Suttiruengwong

**Affiliations:** 1 Department of Materials Science and Engineering, Faculty of Engineering and Industrial Engineering, Silpakorn University, Nakhon Pathom 73000, Thailand; khwanpi37@gmail.com; 2 Department of Physics, Faculty of Science, Silpakorn University, Nakhon Pathom 73000, Thailand; manus_sc.su@hotmail.com

**Keywords:** poly (l-lactic acid), poly (d-lactic acid), nucleating agents, crystallization

## Abstract

The improvement of the rate of crystallization and crystallinity of poly (l-lactic acid) (PLLA) is one of the key performance elements for PLLA to perform better at the higher temperature than its heat deflection temperature (around 60 °C). The organic nucleating agent compounds are one of the interesting choice as they can offer the clarity of products. On the other hand, the nucleated PLLA can be prepared using a low molecular weight poly (d-lactic acid) (PDLA). The aim of this work was to explore the effect of an unsaturated amide compound and PDLA as single and mixed nucleating agents used for PLLA. The crystallization rate and kinetics were investigated and compared for the synthetic unsaturated amide compound (*N*,*N*′-ethylenebis (10-undecenamide) (EBU)) and commercial hydrazide compound (tetramethylenedicarboxylic dibenzoylhydrazide (TMC-306)). PLLA samples was prepared by melt-mixing with TMC or EBU incorporated with peroxide. The influence of different nucleating agents loading on thermal properties, crystallization behaviors, and rheological properties of PLLA were explored by differential scanning calorimetry (DSC) and dynamic mechanical analysis (DMA). The results showed that the addition of EBU or TMC 0.5 phr could pronouncedly increase the crystallinity of PLLA from 3.80% to 24.84% and 8.61%, respectively. The crystallization peak appeared at 112.3 °C in the cooling scan at the rate 7 °C/min when addition EBU and peroxide into PLLA. This indicated that EBU acted as an efficient nucleating agent for PLLA. In isothermal crystallization run at 110 °C, it was found that the overall crystallization rate of nucleated PLLA with TMC or EBU was much faster than neat PLLA. The crystallization half-time indicated that the existence of TMC or EBU could slightly decrease to 2.90 and 1.96 min, respectively compared to neat PLLA (4.60 min). Finally, a low molecular weight PDLA with different contents between 3 and 7 wt % was added in PLLA with EBU and peroxide to investigate the effect of mixed nucleating agents. The crystallization rate of the incorporation of PDLA/EBU/peroxide into PLLA was discussed with the proposed crystallization mechanism. The results revealed the stereocomplex temperature peak at 207 °C as well as normal melting temperature of PLLA. The kinetics of growth crystallization, the crystallization half-time of PLLA at 110 °C was reduced from 4.60 min to 1.96 min (when adding EBU alone) and to 2.62 min (when using mixed PDLA and EBU).

## 1. Introduction

Poly (l-lactic acid) or PLLA is recognized as one of the popular biodegradable polymer. They are derived from renewable resources and possess good mechanical properties and processability. Their products can be formed by a broad spectrum of process technologies including injection molding, fiber spinning, blown film extrusion, thermoforming, and film casting for various applications of biomedical and agricultural materials. [1]. The improvement of PLLA properties is the main interest for further applications in the present. PLLA is a semi-crystalline polymer and its thermal properties and mechanical properties rely on the level of the crystallinity. This has been recognized as a major drawback of PLLA, due to its low crystallization rate and crystallinity. To this point, PLLA processed in a general condition is likely to be an amorphous polymer, hence limiting its use in high temperatures above the glass transition temperature. The solution of this problem is to improve PLLA’s level of crystallinity [1,2].

Harris A.M. et al. [3] reported that neat PLLA annealed at 80 °C for 60 min had the highest crystallinity. The flexural modulus and flexural strength were increased when annealed for 20 min. During processing, the mold temperature was selected to solve this problem. Zhang X. and colleagues [4] showed that s mold temperature higher than the glass transition temperature could improve the crystallinity of PLLA, but the most efficient method to increase the crystallization rate and crystallinity is the use of a nucleating agent. There are many nucleating agents reported for PLLA. Talcum is often chosen because it is inexpensive and can be used as a reinforcement for PLLA [3,5]. Talcum reduces the crystallization half-time of PLLA from 38.2 min to 0.6 min at 115 °C [3]. Alternatively, Nagarajan V. and his group [6] investigated the use of an organic aromatic sulfonate compound (LAK) as a nucleating agent for PLLA The addition of 0.25 wt % LAK could increase the crystallinity of PLLA from 10% (neat PLLA) to 45%. The commercial TMC-306 (TMC) is also a traditional hydrazide compound that can enhance the crystallinity of PLLA [7]. An amide compound is another choice for PLLA nucleation as the amide compound consists of –NH group that can form the hydrogen bond with carbonyl group on PLLA chain, leading to more crystallinity [8]. Nanthananon P. and colleagues [9] found that unsaturated *N*,*N*′-(l,3-propylene) bis (10-undecenamide) (PBU) together with peroxide could improve the rate of crystallization and enhance the crystallinity of PLLA. In addition, poly (d-lactic acid), stereoisomer of PLLA, can be used to form the high melting temperature crystal or stereocomplex crystal (sc-crystals), leading to improved mechanical properties of PLLA [4,10]. Some researchers reported that PDLA acted as a nucleating agent for PLLA [4,11]. Stereocomplex crystals have higher melting temperature (around 210 °C) than that of homocrystal (160–180 °C).

Many researchers reported that several nucleating agents could efficiently enhance the level of crystallinity and the rate of crystallization of PLLA. However, the effect of mixed nucleating agents, unsaturated aliphatic amide compound, and PDLA on the crystallization rate and the crystallinity of PLLA has not yet been reported. In this work, two kinds of aliphatic amide, commercial TMC-306 or synthesized EBU were introduced as a single nucleating agent system while PDLA was further added for the mixed nucleating agent system. Thermal and rheological properties and crystallization behaviors were evaluated by means of the differential scanning calorimetry (DSC), dynamic mechanical analysis (DMA), and wide angle X-ray diffraction (WAXD) respectively.

## 2. Materials and Methods

### 2.1. Materials

**Figure 1:**
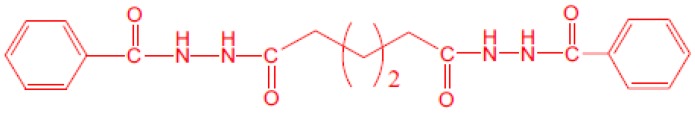
The structure of TMC-306 (hydrazide compound) [7].

**Figure 2:**
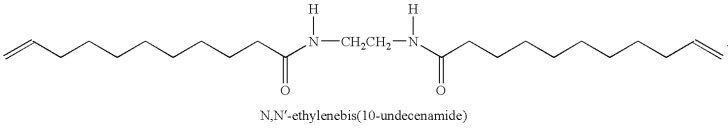
The structure of as synthesized EBU [12].

PLLA extrusion grade (LX-175) consists of 96% l-isomer contents, having melt flow index of 11 g/10 min, and a low molecular weight PDLA (D070) consists of 99% d-isomer contents were kindly provided by Total Corbion PLA Co. Ltd. (Rayong, Thailand). The hydrazide compound as shown in Figure 1 (TMC-306) was kindly supplied by Shanxi Provincial Institute of the Chemical Industry, China. Unsaturated N’N Ethylene bis(10-undecenamide) (EBU) (Figure 2) was synthesized following the method described by Brennen D.J. [12]. The melting temperature and crystallization temperature of EBU was found to be 144.9 °C and 140 °C, respectively.

### 2.2. Sample Preparations

PLLA resins and nucleating agents were dried at 60 °C for 24 h and then blended in internal mixer (MX105-D40L50, Charoentut Model, Charoentut, Co. Ltd., Samutprakarn, Thailand) at 190 °C and 60 rpm for 10 min. Table 1 showed contents of each nucleating agents added in PLLA. After mixing, PLLA samples were compressed at 190 °C for 5 min. (preheat for 4 min and 1 min compression time) to obtain 2 mm thickness sheet samples. The sheet samples were then cut using a laser cutter to form standard specimens for dynamic mechanical analysis (a disc with the diameter of 25 mm) and for wide angle X-ray diffraction (WAXD) (a rectangular sheet of 40 mm × 50 mm).

### 2.3. Characterization

#### 2.3.1. Differential Scanning Calorimetry

DSC measurements were carried out using Perkin Elmer Pyris I instrument under N_2_ atmosphere. Each samples of used about 5–10 mg. In this study, there are two program for measured thermal characteristic and crystallization behaviors respectively.

Program A: For measure thermal characteristics of each samples. The samples were heated from 30 to 190 °C at the heating rate of 7 °C/min and hold for 1 min to delete the prior thermal history. Afterwards, it was cooled to 30 °C at a cooling rate of 7 °C/min and reheated to 190 °C at 7 °C/min.

Program B: For study of crystallization behaviors in an isothermal crystallization process, each sample was heated from 30 to 190 °C at the heating rate of 7 °C/min and hold at final temperature for 5 min to guarantee a totally amorphous state, and then rapidly cooled at a cooling rate 35 °C/min to crystallization temperatures (T_c_) of 95, 100, 105, 110, and 115 °C.

#### 2.3.2. WAXD Measurement

WAXD specimens were cut by a laser cutter. The size of each specimen was 40 mm × 50 mm × 2 mm. The specimens were analyzed with an X-ray diffractometer (LabX XRD-6100, Shimadzu, Bare Scientific Co. Ltd., Bangkok, Thailand) using Cu Kα radiation source (40 kV, 25 mA) and the angle range of 2θ = 5–50° at the scanning rate of 12°/min.

#### 2.3.3. Rheological Measurement

Rheological measurements were accomplished by Anton Paar rheometer (MCR 302, Anton Paar (Thailand) Ltd., Bangkok, Thailand) with parallel plate configuration (diameter of 25 mm). The oscillatory frequency sweep were carried out from 0.1 to 100 Hz at 190 °C with 1% strain for all samples.

## 3. Results

### 3.1. Crystal Behaviors of PLLA with PDLA 

**Figure 3:**
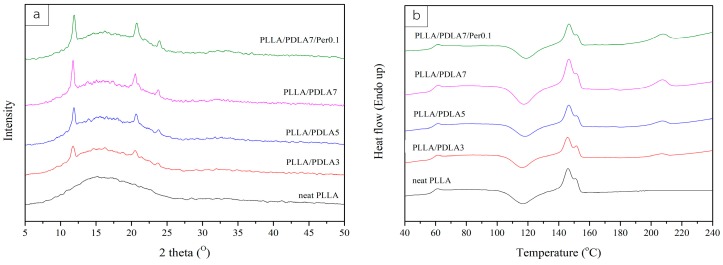
WAXD patterns (**a**) and DSC thermograms of the second heating scan (**b**) of PLLA at different contents of PDLA.

**Figure 4:**
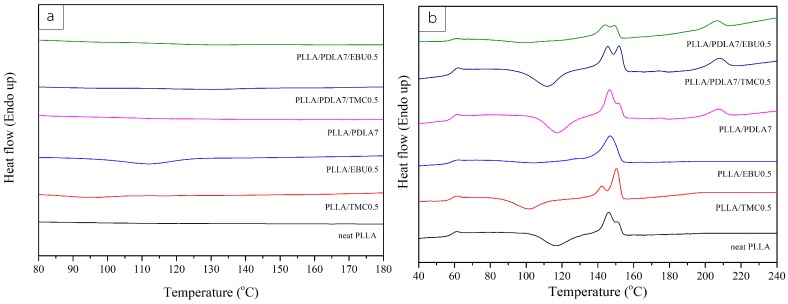
DSC cooling scan (**a**) and DSC second heating scan (**b**) of nucleated PLLA.

The effect of PDLA contents on PLLA was discussed in this section. WAXD and DSC results of PLLA incorporated with PDLA at various contents were shown in Figure 3a,b respectively. The diffraction patterns of sc-crystals were observed at 11.78°, 20.5°, and 23.72° which corresponded with crystal plane (110), (300)/(030), and (220) respectively [4,7,11]. Higher contents of PDLA led to an increased intensity of diffraction peaks. These patterns were not encountered for the diffraction peak of homo-crystal of PLLA, which independently appeared at 16° and 19° [9]. In other words, the introduction of PDLA into PLLA matrix did not influence the occurrence of the homo-crystal structure. DSC measurements were operated by heat–cool–heat scan in the range of temperature between 30 and 250 °C, where the rate of heating and cooling of was fixed at 7 °C/min. In the second heating step, the cold crystallization temperature (T_cc_) appeared at 115–117 °C and the enthalpy of cold crystallization was similar to the enthalpy of homo-crystal melting. The crystallization in the cooling step was not observed in all cases, thus indicating that in such conditions PDLA did not act as a nucleating agent for PLLA. However, sc-crystal melting peak appeared at 205–210 °C and the enthalpy of sc-crystal was greater when increasing the contents of PDLA as illustrated by Figure 4b. The results obtained using DSC analysis were consistent with WXRD study. In addition, a small amount of peroxide was added to PLLA incorporated with PDLA in order to study the effect of the free radical reaction on the crystal structure. This was to ensure that the crystallization was unaffected by the addition of peroxide, which was added when using EBU as a nucleating agent. Based on the WXRD and DSC analysis as shown in Figure 3, the WARD pattern of PLLA/PDLA7/Per0.1 was similar to those without peroxide addition. Similarly, thermal properties did notably not change.

### 3.2. Thermal Characteristics of Nucleated PLLA

The nucleated PLA was referred to PLLA incorporated with a single and mixed nucleating agent system. In Figure 4, the second heating scan of neat PLLA and nucleated PLLA was illustrated. The data of thermal characteristics including the glass transition temperature (T_g_), crystallization temperature (T_c_), melting temperature (T_m_), and cold-crystallization temperature (T_cc_), as well as the enthalpy of melting (∆H_m_) and cold crystallization (∆H_cc_) for all samples are tabulated in Table 2. The percent of crystallinity is evaluated by Equation (1)

(1) $$X_{c}\left(\%\right)=\frac{\Delta H_{m}-\Delta H_{\mathrm{cc}}}{\omega_{\mathrm{PLLA}}\Delta H_{m}^{0}}\times 100$$

where ω_PLLA_ is the weight fraction of PLLA, and $\Delta H_{m}^{0}$ is the enthalpy of total crystalline melting of PLLA (taken to be 93.1 J/g) [13].

Table 2 presents thermal parameters from the second heating scan. In a single nucleating system, amide compound (EBU) and hydrazide compound (TMC-306) were compared. The addition of 0.5 wt % TMC-306 in PLLA hardly affected to the degree of crystallinity of PLLA when compared with the addition of 0.5 wt % EBU (peroxide was added to promote the free radical reaction of double bonds in EBU compounds). Our previous work [9] investigated the influence of the reactive aliphatic bisamide on the crystallization enhancement of PLLA. *N*,*N*′-(l,3-propylene) bis(10-undecenamide) (PBU), which has two double-bonds chemical structure as well as synthesized EBU in this work. It was found that the addition of peroxide promoted the fast crystallization rate due to the crosslinked structure and induced crystallization [9]. The chemical structure of EBU consists of two double bonds at its moiety. These functional groups can react with peroxide to form free radical and form the polymerized EBU, grafted, or crosslinked structures of PLLA or even PDLA. This structure allows PLLA to crystalline more easily. Without the addition of peroxide, the crystallization behavior of PLLA was unaffected. The degree of crystallinity of PLLA was increased from 3.80 % (neat PLLA) to 24.94% for PLLA/EBU0.5 and 8.61% for PLLA/TMC0.5. EBU was more effective nucleating agent than TMC. The reason for this may be due to EBU grafted onto PLLA structure. Nanthananon P. and coworkers [7] studied the reactive aliphatic bisamide compound as a potential nucleating agent. They synthesized *N*,*N*′-(1,3-propylene) bis(10-undecenamide) (PBU) and found that the unsaturated structure could react with peroxide to form grafted PLLA structure, leading to a higher crystallization rate and crystallinity [9]. In our case, EBU grafted PLLA might serve as in situ starting nucleation site for PLLA. TMC-306 and EBU could reduce the cold-crystallization temperature of PLLA and increase the degree of crystallinity at the cooling step. These results indicated that both EBU and TMC-306 acted as nucleating agents for PLLA. The addition of 7 wt % PDLA into either EBU or TMC-306 was hardly increased the degree of crystallinity of PLLA. The cold-crystallization temperature remained almost unchanged. PDLA was reported to be a nucleating agent for PLLA with homo-polymer grade (<99% l-lactic acid) from the literature [4]. The loading of only 5 wt % PDLA increased the crystallinity of PLLA to 37.7%. However, this was not in our case, where PLLA grade 96% L-lactic acid was selected.

In the case of mixed nucleating agents, the amide compound or hydrazide compound together with PDLA was incorporated in PLLA matrix. The degree of crystallinity of PLLA incorporated with TMC-306 and PDLA did not change when compared to the addition of TMC-306 alone. The melting enthalpy of sc-crystal was as high as 7.47 J/g whereas for PLLA incorporated with EBU and PDLA, the enthalpy of the homo-crystal was decreased and the cold crystallization temperature (T_cc_) was decreased to 98.5 °C when compared with addition of EBU alone. The reduced T_cc_ was the result of the existence of incompletely crystallization that occurred in the cooling scan. When the polymer was heated, the incomplete crystals acquired the energy to reorganize and form the complete homo-crystal structure. It occurred more easily than the system which was not incompletely crystal. However, the degree of crystallinity was less than the single EBU nucleating system. This finding suggested that two separate forms of crystals occurred when using mixed nucleating agent system The addition of PDLA caused the reduction of the percent of crystallinity of PLLA (%X_c_). Taking %X_c_ into account, the mixed nucleating agent system was less effective than the single EBU nucleating agent system. The incorporation of PDLA in mixed nucleating agent system decreased the percent of crystallinity of PLLA.

### 3.3. Crystal Structures of Nucleated PLLA

**Figure 5:**
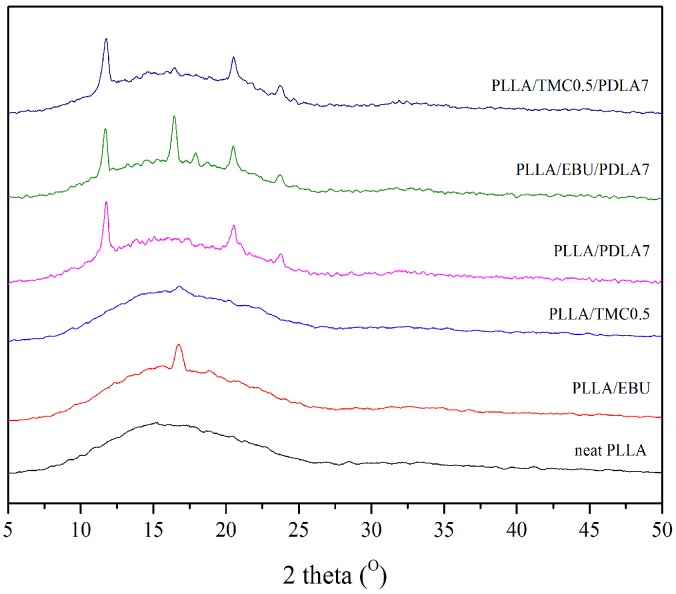
WAXD patterns of nucleated PLLA.

Figure 5 showed WAXD patterns of nucleated PLLA, a single EBU nucleating agent system displayed the strong homo-crystal peak 2θ at 16.4° but this peak was weak for the single TMC nucleating agent system. The homo-crystal peak corresponds to α crystal form of PLLA. In fact, PLLA has two crystal structures—α form and α′ form. The α form has tight arrangement of crystal structure and the α′ form has loose arrangement of crystal structure [1]. In the case of the mixed nucleating agents system, they showed 2θ peaks of sc-crystal peak at 11.7°, 20.5°, 23.7° and homo-crystal peak at 16.4° and 17.9°. This result suggested that the addition of PDLA could lead to sc-crystal form in PLLA and had an effect on different crystal structures in nucleated PLLA.

### 3.4. Rheological Properties

**Figure 6:**
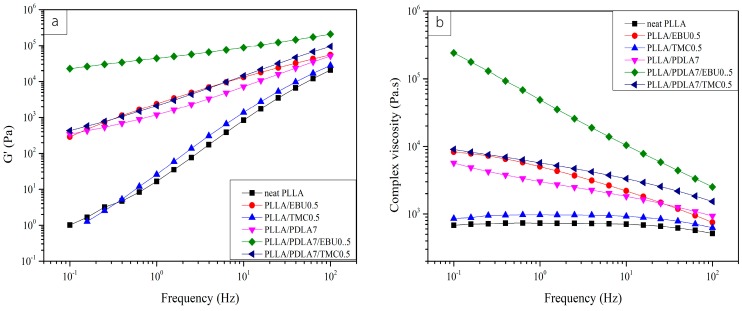
Storage modulus (**a**) complex viscosity (**b**) at various frequency for nucleated PLLA.

To explore the effect of nucleating agents, both a single and mixed system, on rheological properties of the nucleated PLLA, the frequency sweep oscillatory shear measurements were performed at 190 °C. It should be noted that, at the studied temperature, the homo-crystal of PLLA was melted whereas sc-crystal still remained in the polymer melt. From Figure 6a, it displayed the storage modulus (G’) and the complex viscosity for neat PLLA and nucleated PLLA at different test frequency. The storage modulus (G’) increased when adding nucleating agents in PLLA, except the addition of 0.5 wt % TMC in PLLA, whose storage modulus was similar to that of neat PLLA as seen in Figure 6a. Neat PLLA exhibited the typical terminal behavior. The slope of the storage modulus curve at low frequency decreased when adding nucleating agents containing PDLA and EBU. In the case of the EBU nucleating agent system, the higher storage modulus might be due to the possible change of the PLLA chemical structure though free radical reaction resulted from the reaction of peroxide and two double bonds in EBU molecules, therefore more elastic in the melts state. In the case of the addition of PDLA and PDLA with TMC, the increase in the elastic modulus in the melt might be due to the physical crosslink though existing sc-crystal [13]. The shear thinning of complex viscosity in the mixed EBU and PDLA system implied the high molecular weight resulted from the chemical and physical crosslinking whereas with single PDLA and mixed PDLA and TMC systems, it showed less effect of shear thinning of complex viscosity, which implied a lesser crosslinking effect.

### 3.5. Isothermal Crystallization Behaviors

Isothermal crystallization behavior of nucleated PLLA prepared using a single and mixed nucleating agents system was examined between 95 and 115 °C, which was this range of the cold crystallization temperature of PLLA as suggested elsewhere [14]. The Avrami theory is the general method for explaining isothermal crystallization kinetics. The Avrami equation is showed below [14]

X_t_ = 1 − exp (−kt^n^), (2)

when k is the rate constant of growth crystallization, and n is the Avrami exponent, which describes the nature of crystal growth. For convenience, the equation is rewritten as Equation (3) and, for easy understanding, the crystallization half-time can be computed by Equation (4)

ln [−ln (1 − X_t_) ] = n lnt + ln k, (3)

t_1/2_ = (ln2/k)^1/n^, (4)

**Figure 7:**
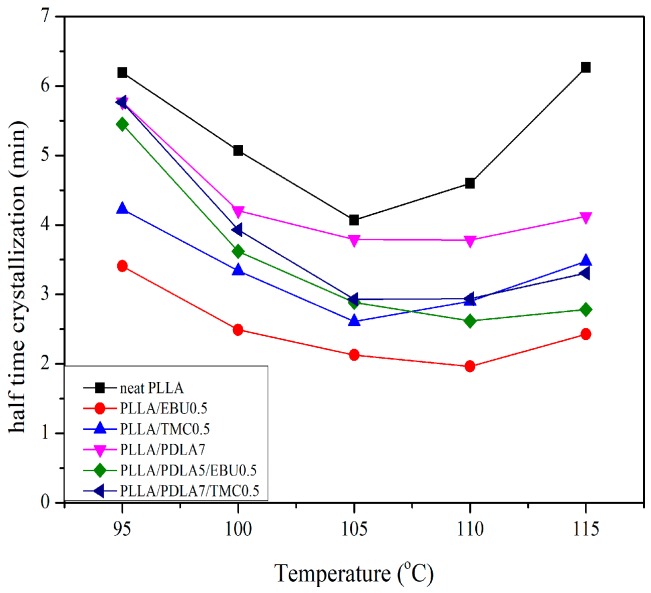
Dependence of half-time crystallization on crystallization temperature of nucleated PLLA.

The calculation results of k, n are included in Table 3. It could be seen that the value of n changed within the range of 2.3–3.8 and the lowest value of n was 2.3 for neat PLLA at 115 °C and the highest value of n is 3.8 for PLLA/TMC0.5 at 115 °C. The growth crystallization half-time could be calculated by Equation (4) and plotted with the crystallization temperature in Figure 7. PLLA/EBU0.5 system showed the lowest growth crystallization half-time, which corresponded to the DSC results of thermal properties. For the kinetics of crystallization growth, the crystallization half-time of PLLA was reduced from 4.60 min to 1.96 min (PLLA and EBU) and to 2.62 min (PLLA with PDLA and EBU) at 110 °C. For the latter case, the slower crystallization half-time could possibly be due to the reaction of EBU and peroxide with low molecular weight PDLA, hence inhibiting PDLA from acting as a nucleating agent for PLLA. More works on this reaction should be performed without peroxide. However, all systems of nucleated PLLA showed the relatively similar growth rate, where the crystallization half-time varied from 2.5 to 4.5 min for the best condition of each system. In a mixed nucleating system, the existence of PDLA and EBU or TMC, the reduced crystallization half-time was observed, but the reduction was still less than the addition of EBU alone.

## 4. Conclusions

The effects of a single EBU incorporated with peroxide or TMC-306, a mixed PDLA and EBU, or PDLA and TMC-306 nucleating agent on crystallization behaviors and crystal structures was evaluated. Either TMC or EBU could act as nucleating agents for PLLA. EBU incorporated with peroxide proved to be a more effective nucleating agent than TMC-306. PDLA could not act as nucleating agent for PLLA in this work, but rather created the sc-crystals in the process as confirmed from DSC and XRD results. The mixed PDLA and TMC or EBU in PLLA could result in the formation of the sc-crystals and homo-crystals of PLLA. This result was clearly pronounced for the mixed PDLA and EBU system. The addition of EBU alone showed the low cool-down crystallinity and the percent of crystallinity increased from 3.8 % (neat PLLA) to 24.94 % while PDLA and EBU system showed an even lower cool-down crystallinity than EBU alone. The crystallization half-time of PLLA at 110 °C were reduced from 4.60 min to 1.96 min (the addition of EBU) and to 2.62 min (the addition of mixed PDLA and EBU). The dynamic mechanical test results revealed that a mixed PDLA and EBU nucleating agents system could promote melt elastic properties as indicated by the increase in storage modulus and shear thinning complex viscosity effect. This suggests that the addition of PDLA can be beneficial when the flow modification is required for processing PLLA.

## Figures and Tables

**Table 1:** PLLA and its compositions prepared by melting mixing equipment.

Sample	Content of Substance				
	PLLA (%)	PDLA (%)	TMC (%)	EBU (%)	Peroxide (phr)
Neat PLLA	100.0	-	-	-	-
PLLA/EBU0.5 *	99.5	-	-	0.5	0.1
PLLA/TMC0.5	99.5	-	0.5	-	-
PLLA/PDLA7	93.0	7.0	-	-	-
PLLA/PDLA7/EBU0.5 *	92.5	7.0	-	0.5	0.1
PLLA/PDLA7/TMC0.5	92.5	7.0	0.5	-	-

* Peroxide of 0.1 phr added to promote the free radical reaction.

**Table 2:** Thermal characteristic of nucleated PLLA as determine by second heating scan.

Sample Name	T_g_ (°C)	T_c_ (°C)	T_cc_ (°C)	T_m_ (°C)	ΔH_m_ (J/g)	ΔH_cc_ (J/g)	X_c_ (%)	T_sc_ (°C)	ΔH_m,sc_ (J/g)
Neat PLLA	58.7	-	117.1	146.1, 149.8	26.37	22.84	3.80	-	-
PLLA/EBU0.5 *	59.1	112.3	102.6	146.7	25.15	2.04	24.94	-	-
PLLA/TMC0.5	58.4	97.5	102.0	142.2, 150.4	25.75	17.74	8.61	-	-
PLLA/PDLA7	59.6	-	117.2	146.6, 150.7	25.82	19.45	7.35	207.0	6.20
PLLA/EBU0.5/PDLA7 *	58.9	-	98.5	144.0, 149.1	19.48	6.87	14.64	206.0	6.66
PLLA/TMC0.5/PDLA7	59.8	-	111.8	145.6, 151.8	25.45	18.05	8.59	207.9	7.47

* Peroxide was added together with EBU.

**Table 3:** Kinetics parameters obtained from the isothermal crystallization of nucleated PLLA.

Sample Name	Temp. (°C)	n	k (min^−1^)	t_1/2_ (min)
Neat PLLA	95	2.5	0.0073	6.19
	100	2.9	0.0063	5.07
	105	2.7	0.0157	4.07
	110	2.5	0.0153	4.60
	115	2.3	0.0102	6.26
PLLA/EBU0.5 *	95	2.7	0.0253	3.41
	100	2.8	0.0539	2.49
	105	3.0	0.0721	2.13
	110	3.1	0.0858	1.96
	115	3.0	0.0486	2.43
PLLA/TMC0.5	95	2.4	0.0218	4.22
	100	2.8	0.0236	3.34
	105	2.6	0.0573	2.61
	110	3.2	0.0229	2.90
	115	3.8	0.0061	3.48
PLLA/PDLA7	95	2.6	0.0073	5.77
	100	2.9	0.0108	4.20
	105	3.0	0.0127	3.79
	110	3.0	0.0128	3.79
	115	2.8	0.0131	4.12
PLLA/PDLA7/TMC0.5	95	2.3	0.0123	5.77
	100	2.5	0.0226	3.93
	105	2.6	0.0425	2.93
	110	3.2	0.0220	2.94
	115	3.3	0.0134	3.31
PLLA/PDLA7/EBU0.5 *	95	2.9	0.0051	5.45
	100	3.1	0.0129	3.62
	105	3.0	0.0289	2.88
	110	3.1	0.0352	2.62
	115	3.2	0.0263	2.78

* PLLA/EBU samples including peroxide.
